# Changes in plasma metabolite concentrations and enzyme activities in aging riding horses

**DOI:** 10.3389/fvets.2024.1345548

**Published:** 2024-05-31

**Authors:** Yukari Asahi, Toshiro Arai, Yoshikazu Tanaka

**Affiliations:** School of Veterinary Medicine, Nippon Veterinary and Life Science University, Musashino, Japan

**Keywords:** adiponectin, age-related disease, chronic inflammation, inflammaging, serum amyloid A

## Abstract

In older horses, basal metabolic rate decreases, and plasma metabolite and hormone concentrations related to energy metabolism change. The occurrence of age-related diseases, which increases in old animals, may enhance inflammatory reactivity (inflammaging). Finding the appropriate treatment for inflammaging at an early stage may prevent various age-related diseases. Changes in metabolite and hormone concentrations and enzyme activities involved in energy metabolism in the plasma of clinically healthy riding horses of various ages were measured to identify biomarkers of inflammaging (persistent low-grade inflammation that occurs with aging). All horses were clinically healthy, and their body condition scores (BCSs) were 4 or 5 (9-point scale). Plasma triglyceride (TG), total cholesterol (T-Cho), blood urea nitrogen (BUN), insulin concentrations, malondialdehyde (MDA), and serum amyloid A (SAA) concentrations generally increased with age. Adiponectin concentrations, plasma superoxide dismutase (SOD), and leukocyte AMP-activated protein kinase (AMPK) activities decreased, while plasma aspartate aminotransferase (AST), alanine aminotransferase (ALT), and glutathione peroxidase (GPx) remained unchanged as horses aged. Although riding horses that partake in continuous exercise seems to be less likely to develop inflammaging, horses over 17 years of age tend to show proinflammatory signs with disordered lipid metabolism. In riding horses, SAA, in combination with other markers, may be a useful biomarker for inflammaging and dysregulated lipid metabolism in aging horses.

## 1 Introduction

The percentage of horses considered “aged” has been increasing, with estimates of all horses older than 15 years of age ranging from 22 to 34% (1, 2). In older horses, basal metabolic rate decreases (3), and plasma metabolite and hormone concentrations related to energy metabolism change with age (4). The occurrence of age-related diseases increases in old animals (5, 6), and enhanced inflammatory reactivity known as inflammaging may arise with their onset (7, 8). Franceschi et al. termed the persistence of low-grade chronic inflammatory status as inflammaging (9), which involves the accumulation of damaged macromolecules and cellular debris because of increased production and chronically inhibited damage surveillance and repair functions in multiple tissues (10). The secretion of proinflammatory cytokines from senescent cells accumulates in tissues with age, termed “senescence-associated secretory phenotype (SASP),” and contributes to the onset of inflammaging (11). As plasma concentrations of macromolecules, metabolites, hormones, and enzymes involved in energy metabolism change in animals with age, monitoring these concentrations can aid in detecting inflammaging. Appropriate treatment for inflammaging at an early stage may prevent various age-related diseases. Arfuso et al. reported that serum C-reactive protein (CRP) and α2 macroglobulin concentrations increased in horses with aging, and acute phase proteins (APPs) such as CRP could be good parameters to assess inflammaging in horses (12). In this study, changes in metabolite and hormone concentrations and enzyme activities involved in energy metabolism in the plasma of clinically healthy riding horses of various ages were measured. Macromolecules that vary with age are considered to be useful biomarkers for diagnosing inflammaging in riding horses.

## 2 Materials and methods

### 2.1 Animals

In this study, 18 riding horses (Thoroughbred, gelding, 2–23 years of age) maintained at Niiza Riding Club (Saitama, Japan) and Saitama Horse Riding Club (Saitama, Japan) were examined. Their body condition score (BCS) was classified using a 9-point system. Six horses were selected for each of three groups based on age: Group A (adolescent, <10 years old), Group B (middle age, 10–16 years old), and Group C (old age, >17 years old). The horses were fed 5.2–6.4 kg of hay cube, 3.0–4.0 kg of good quality hay, 0–1.3 kg of wheat bran, and 0–1.8 kg of barley at 6:00 and 16:00 daily. Each horse was subjected to exercise, which included walking (2–3 m/s for 5–10 min) and trotting (4–6 m/s for 15–20 min) every day of the week, resting on Sunday, over a 10-week period.

### 2.2 Collection and preparation of blood samples

Blood samples were taken from the jugular veins of horses and placed in heparinized tubes. Plasma was then recovered by centrifugation at 800 × g for 10 min at 4°C and stored at −25°C until needed for further analysis. Leukocytes were isolated by centrifugation using hypotonic and hypertonic solutions. Cytosolic fractions of leukocytes were prepared and isolated according to the method previously described (13).

### 2.3 Plasma metabolite and hormone assay

Plasma glucose, triglyceride (TG), total cholesterol (T-Cho), total protein (TP), creatinine, and blood urea nitrogen (BUN) concentrations were measured using an automatic clinical chemistry analyzer (BioMajesty™ JCA-BM2250, JEOL Ltd., Tokyo, Japan) with the manufacturer reagents at FUJIFILM VET Systems Co., Ltd. (Tokyo, Japan). Non-esterified fatty acids (NEFAs) and malondialdehyde (MDA) concentrations were measured using NEFA C-test Wako (FUJIFILM Wako Pure Chemical Corporation, Osaka, Japan) and NWLSS™ Malondialdehyde Assay (Northwest Life Science Specialties, LLC, WA, USA) commercial kits, respectively. Serum amyloid A (SAA) concentrations were measured using a biochemistry automatic analyzer (Hitachi 7,180, Hitachi High-Tech Corporation, Tokyo, Japan) with a commercial kit, VET-SAA (EIKEN CHEMICAL CO., LTD., Tokyo, Japan). Insulin and adiponectin concentrations were measured using LBIS Rat Insulin ELISA kit (FUJIFILM Wako Pure Chemical Corporation, Osaka, Japan) and Mouse/rat adiponectin ELISA kit (Otsuka Pharmaceutical Co., Ltd., Tokyo, Japan), respectively. Insulin and adiponectin concentrations were measured by the ELISA analysis (14), and 100 times diluted plasma was used for the adiponectin assay. All calibrators and samples were run in duplicate, and the samples exhibited parallel displacement to the standard curve for the ELISA analysis. The intra- and inter-assay coefficients of variation were at <10% in both insulin and adiponectin assay.

### 2.4 Plasma enzyme activity assay

Aspartate aminotransferase (AST) and alanine aminotransferase (ALT) activities were measured using an automatic clinical chemistry analyzer (BioMajesty™ JCA-BM2250, JEOL Ltd., Tokyo, Japan) with the manufacturer’s reagents at FUJIFILM VET Systems Co., Ltd. (Tokyo, Japan). Glutathione peroxidase (GPx) and superoxide dismutase (SOD) activities were measured using commercial kits, GPx activity assay (Northwest Life Science Specialties, LLC, WA, USA) and SOD assay kit-WST (DOJINDO LABORATORIES, Kumamoto, Japan), respectively. Malate dehydrogenase (MDH) and lactate dehydrogenase (LDH) activities were measured using the methods previously described (15, 16). The M/L ratio was calculated as MDH activities divided by LDH activities. A higher M/L ratio reflects elevated energy metabolism, leading to higher ATP production in some tissues, such as muscle and the liver (17). AMP-activated protein kinase (AMPK) activities in the cytosolic fraction of peripheral leukocytes were measured using a commercial kit (CycLex AMPK Assay Kit, MEDICAL & BIOLOGICAL LABORATORIES CO., LTD., Tokyo, Japan). The protein concentration in the cytosolic fraction was determined by the Bradford method (18).

### 2.5 Statistical analysis

The measured values are expressed as a means±standard error (SE). Statistical significance was determined by Student’s *t*-test. The significance level was set at a *p*-value of <0.05. Measured values outside the reference range were excluded.

## 3 Results

All horses were clinically healthy, and their BCS values were 4 or 5 on the 9-point scale. Plasma glucose, TP, creatinine, and NEFA concentrations in horses remained unchanged through adolescence, middle age, and old age. TG, T-Cho, BUN, and insulin concentrations increased with age, while adiponectin concentrations decreased. MDA and SAA concentrations did not change between adolescence and middle age but increased with aging beyond middle age. The MDA concentrations in the old age group were significantly higher than those in the adolescent group (Table 1). Plasma AST, ALT, and GPx activities did not change in the horses with age, while plasma SOD and leukocyte AMPK activities decreased. The plasma M/L ratio did not change with aging (Table 2).

**Table 1:** Changes in plasma metabolite and hormone concentrations in aging riding horses.

		Group A (*n* = 6) adolescent, <10 years old	Group B (*n* = 6) middle age, 10–16 years old	Group C (*n* = 6) old age, >17 years old
Glucose	(mg/dL)	90 ± 3	92 ± 2	90 ± 2
TG	(mg/dL)	14 ± 2	18 ± 3	20 ± 3
T-Cho	(mg/dL)	77 ± 7	79 ± 6	95 ± 5
NEFA	(μEq/L)	89 ± 12	98 ± 20	82 ± 7
TP	(g/dL)	5.9 ± 0.2	6.1 ± 0.2	5.9 ± 0.1
Creatinine	(mg/dL)	1.2 ± 0.1	1.2 ± 0.1	1.2 ± 0.0
BUN	(mg/dL)	16 ± 1	19 ± 1	20 ± 1
MDA	(μmol/L)	7.6 ± 2.5	7.6 ± 2.5	13.8 ± 0.9 *
SAA	(mg/L)	1.4 ± 0.4	0.6 ± 0.1	2.3 ± 1.3
Insulin	(ng/mL)	0.9 ± 0.4	1.0 ± 0.5	1.4 ± 0.8
Adiponectin	(μg/mL)	9.1 ± 2.0	8.1 ± 1.5	7.1 ± 1.1

*Significant differences (*p* < 0.05) from the value in Group A (Student’s *t*-test).

**Table 2:** Changes in plasma enzyme activities in riding horses with aging.

		Group A (*n* = 6)	Group B (*n* = 6)	Group C (*n* = 6)
AST	(U/L)	221 ± 18	226 ± 18	253 ± 25
ALT	(U/L)	7.0 ± 0.9	6.8 ± 0.6	7.5 ± 1.2
GPx	(U/L)	5.5 ± 1.9	5.9 ± 0.8	5.4 ± 0.3
SOD	(unit/mL)	114 ± 29	97 ± 11	94 ± 13
MDH	(U/L)	152 ± 12	155 ± 18	167 ± 15 *
LDH	(U/L)	215 ± 28	214 ± 20	246 ± 37
M/L	Ratio	0.72 ± 0.05	0.73 ± 0.04	0.70 ± 0.04
AMPK**	(μg/min/mg)	1.4 ± 0.3	1.3 ± 0.3	1.0 ± 0.1

A: <10 years B: 10–16 years C: >17 years. *Significant differences (*p* < 0.05) from the value in Group A (Student’s *t*-test). **AMPK activities were measured in the cytosol of peripheral leukocytes.

## 4 Discussion

According to their plasma ALT and AST activities and creatinine and BUN concentrations, it could be presumed that no riding horses in this study had any specific lesions in their livers, kidneys, or skeletal muscles. Although the BCS of riding horses did not increase with aging, they showed symptoms of insulin resistance, including increases in both plasma insulin and TG. In healthy, middle-aged humans, insulin resistance and dyslipidemia associated with the accumulation of excess visceral adipose tissue are frequently observed (19, 20). A horse at 17 years of age is equivalent to a human at 50 years of age (21). Dysregulation of lipid metabolism became clear in riding horses aged over 17 years. The increases in plasma MDA and SAA concentrations and decreases in SOD activities were notable. White adipose tissue dysfunction and the accumulation of visceral fat occur in elderly humans (22, 23). Although older riding horses did not show marked obesity, they accumulated more dysregulated adipocytes (enlarged adipocytes) as visceral fat compared to younger horses (4). In older riding horses, plasma adiponectin concentrations decreased, as is the case for aging humans. The molecular mechanisms responsible for adiponectin concentrations may be the direct result of inflammatory cells suppressing reactive oxygen species (ROS) and cytokines, inhibiting the NF-κB inflammatory signaling pathway, and downregulating inflammatory responses (24, 25). Decreasing adiponectin concentrations lead to dyslipidemia and insulin resistance (26). Obesity is known to affect immunity and inflammation in horses, and failure to control for BCS can obscure the interpretation of these results (27, 28). Increased plasma SAA concentrations in obese animals could be the result of normal fat mass and/or increased expression and secretion of SAA from dysfunctional adipose tissue or other tissues (26). Concentrations of MDA as a product of lipid peroxidation (29) increase with chronic inflammation as a result of ROS. Increased MDA in older riding horses is considered to be induced by lower adiponectin concentrations and decreased SOD activities.

SAA from dysfunctional adipose tissue may act locally to alter cytokine production and fat metabolism. Additionally, it may act systemically on the liver, muscle, cells of the immune system, and the vasculature to impact insulin resistance and atherosclerosis (26). Although SAA is commonly used as a marker for APP in cats and horses (30, 31), it may be used as a marker of inflammaging in riding horses when combined other lipid metabolism markers.

Although riding horses with sufficient exercise continually seem to be less likely to develop inflammaging, horses older than 17 years tend to show a proinflammatory status with dysregulated lipid metabolism. Current SAA reagents do not have enough sensitivity to confirm a significant difference; however, it is expected that improved reagent performance will allow SAA to become a biomarker that can differentiate the presence or absence of inflammaging with dysregulated lipid metabolism in riding horses.

This study has some limitations. First, the number of horses in each group was small. Second, the insulin, adiponectin, and SAA antibodies for measurements were not specific to the equine species. An age- and sex-matched control group, fed on a similar diet, would be required to help eliminate the possible influence of the aforementioned factors. Further studies should be performed to evaluate the usefulness of SAA and other macromolecules such as TG, NEFA, MDA, and SOD as biomarkers for diagnosing inflammaging in riding horses.

## 5 Conclusion

Changes in metabolite and hormone concentrations and enzyme actiMalate dehydrogenase UV assayities relating to energy metabolism in the plasma of clinically healthy riding horses of various ages were measured. All horses had BCS values of 4 or 5, without obesity. Plasma TG, T-Cho, BUN, and insulin concentrations increased gradually with aging, whereas plasma adiponectin concentrations decreased. MDA and SAA concentrations increased with aging, while plasma AST, ALT, and GPx activities did not change. Furthermore, plasma SOD and leukocyte AMPK activities decreased. Although riding horses with sufficient and continuous exercise seem to be less likely to develop inflammaging, horses over the age of 17 years tend to show a proinflammatory status with the disorder of lipid metabolism. SAA, in combination with other lipid metabolism markers, may be a useful biomarker for inflammaging with dysregulated lipid metabolism in riding horses.

## Data availability statement

The original contributions presented in the study are included in the article/supplementary material, further inquiries can be directed to the corresponding author.

## Ethics statement

The animal study was approved by the Nippon Veterinary and Life Science University Animal Research Committee. The study was conducted in accordance with the local legislation and institutional requirements.

## Author contributions

YA: Writing – original draft, Validation, Resources, Methodology, Investigation, Formal analysis, Data curation, Conceptualization. TA: Writing – review & editing, Validation, Supervision, Project administration, Formal analysis, Data curation, Conceptualization. YT: Writing – review & editing, Validation, Resources, Methodology, Data curation.
